# Dual‐targeting therapy against HER3/MET in human colorectal cancers

**DOI:** 10.1002/cam4.5673

**Published:** 2023-02-07

**Authors:** Akitaka Yamasaki, Rikuto Miyake, Yuta Hara, Hideki Okuno, Takuya Imaida, Kouki Okita, Shogo Okazaki, Yasutoshi Akiyama, Kenji Hirotani, Yuichi Endo, Kazue Masuko, Takashi Masuko, Yoshihisa Tomioka

**Affiliations:** ^1^ Cell Biology Laboratory, Faculty of Pharmacy Kindai University Osaka Japan; ^2^ Laboratory of Oncology Pharmacy Practice and Science, Graduate School of Pharmaceutical Sciences Tohoku University Sendai‐shi Japan; ^3^ Production and Manufacturing Carna Biosciences, Inc. Kobe Japan; ^4^ Division of Immunology and Pathobiology, Department of Microbiology Nihon University School of Density Tokyo Japan; ^5^ Early Clinical Development Department, R&D Division Daiichi Sankyo Co., Ltd. Tokyo Japan; ^6^ Natural Drug Resources, Faculty of Pharmacy Kindai University Osaka Japan

**Keywords:** colorectal cancer, Forkhead Box protein M1, human epidermal growth factor receptor family, mesenchymal to epithelial transition factor

## Abstract

**Background:**

Colorectal cancer (CRC) is the most common malignancy in the world, and novel molecular targeted therapies for CRC have been vigorously pursued. We searched for novel combination therapies based on the expression patterns of membrane proteins in CRC cell lines.

**Results:**

A positive correlation was observed between the expression of human pidermal growth factor receptor (HER) 3 and mesenchymal‐to‐epithelial transition factor (MET) on the cell surface of CRC cell lines. The brief stimulation of HER3/MET‐high SW1116 CRC cells with both neuregulin‐1 (NRG1) and hepatocyte growth factor enhanced ERK phosphorylation and cell proliferation more than each stimulation alone. In addition, a prolonged NRG1 stimulation resulted in the tyrosine phosphorylation of MET. In this context, the Forkhead Box protein M1 (FOXM1)‐regulated tyrosine phosphorylation of MET by NRG1 was demonstrated, suggesting the existence of a signaling pathway mediated by FOXM1 upon the NRG1 stimulation. Since the co‐expression of HER3 and MET was also demonstrated in in vivo CRC tissues by immunohistochemistry, we investigated whether the co‐inhibition of HER3 and MET could be an effective therapy for CRC. We established HER3‐and/or MET‐KO SW1116 cell lines, and HER3/MET‐double KO resulted in the inhibition of in vitro cell proliferation and in vivo tumor growth in nude mice by SW1116 cells. Furthermore, the combination of patritumab, an anti‐HER3 fully human mAb, and PHA665752, a MET inhibitor, markedly inhibited in vitro cell proliferation, 3D‐colony formation, and in vivo tumor growth in nude mice by SW1116 cells

**Conclusion:**

The dual targeting of HER3/MET has potential as CRC therapy.

## Graphical abstract

The present study demonstrated that HER3 and MET were not directly associated with each other in CRC but cooperated for the cellular and tumor growth of CRC. In this context, the combination of anti‐HER3 (patritumab) and anti‐MET (PHA665752) drugs was definitely effective against HER3/MET‐high SW1116 CRC cells, indicating the potential of the dual inhibition of HER3 and MET as targeting therapy in human CRC.

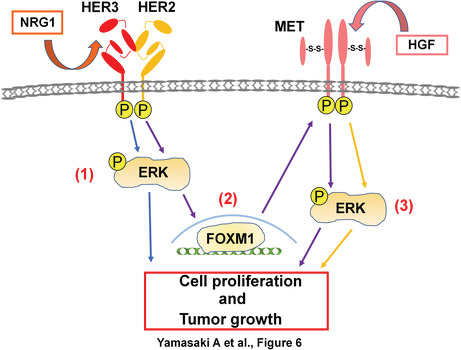

## 1 INTRODUCTION

Colorectal cancer (CRC) is one of the leading causes of human cancer death worldwide.^1^, ^2^ Surgery and classical chemotherapy are generally the main treatment for CRC. Furthermore, molecular targeted therapy against human epidermal growth factor receptor (HER) 1 with therapeutic monoclonal antibodies (mAb) such as cetuximab and panitumumab are effectively administered to patients with CRC,^3^ and other HER family proteins may also be attractive targets for CRC therapy. The HER family consists of four receptor‐type tyrosine kinases: HER1 (EGFR), HER2 (*erb*B‐2), HER3 (*erb*B‐3), and HER4 (*erb*B‐4), and these receptors form homo‐ or heterodimers, leading to the activation of receptors. The activation of HER family proteins stimulates intracellular events, such as the MAPK and PI3K‐AKT pathways,^4^ resulting in cancer initiation and progression.

The phosphor‐transfer reaction of HER3 is catalytically impaired, resulting in very weak kinase activity; however, HER3 may be activated by forming heterodimers with other HER family receptors. By the binding to HER3 ligands, like neuregulin 1 (NRG1), HER3 plays important roles, such as in the differentiation and proliferation of normal and cancer cells.^5^, ^6^, ^7^, ^8^ The kinase activity of the HER2/HER3 heterodimer is stronger than that of other HER family heterodimers.^9^, ^10^ Regarding molecular targeted cancer therapies, HER3 was found to be crucial for limiting the effects of HER kinase inhibitors.^11^, ^12^

Mesenchymal‐to‐epithelial transition factor (MET or c‐MET) is a receptor‐type tyrosine kinase that is activated by the binding of hepatocyte growth factor (HGF). The HGF/MET pathway is essential in cell proliferation, motility, and the development of resistance to cancer therapy.^13^, ^14^

Growing evidence has suggested that the presence of different cell populations within a single tumor may limit the effectiveness of therapies targeting a single molecule.^15^, ^16^ Therefore, the efficacy of cancer therapy may be enhanced by simultaneously inhibiting multiple therapeutic targets. We searched for novel combination therapies based on an expression analysis of membrane proteins on CRC cell lines.

In the present study, we demonstrate that the cell surface expression of HER3 and MET proteins positively correlates in human CRC, the NRG1‐stimulated tyrosine phosphorylation of MET is regulated by the transcription regulator, Forkhead Box protein M1 (FOXM1), and the co‐inhibition of HER3 and MET represents an effective therapy for human CRC.

## 2 MATERIALS AND METHODS

### 2.1 Animals

Female F344 rats and Male KSN nude mice were obtained from Shimizu Animal Farm (Kyoto, Japan). Animals were individually raised in plastic cages and maintained under specific pathogen‐free conditions and housed in under a standard light/dark cycle at a constant temperature of 23 ± 1°C. Experiments were approved by the Committee for the Care and Use of Laboratory Animals at Kindai University (KAPS‐23‐004) and performed following the institutional guidelines and the United States National Institutes of Health Guide for the Care and Use of Laboratory Animals.

### 2.2 Cell culture

The following 23 human CRC cells (SW1116, LS174‐T, LS180, SW480, RKO, RKO‐E6, WiDi, LS123, CCK81, HCC56, SW620, SW1417, LoVo, HT29, LS1034, DLD‐1, Caco2, HCT15, SNU‐C1, T84, OUMS‐23, Colo201, and HCT116), and P3X63Ag8.653 mouse myeloma cells were obtained from the American Type Cell Collection (Manassas, VA, USA). CCD841 non‐neoplastic colon cell line was obtained from the Japanese Collection of Research Bioresources Cell Bank. The RH7777 rat hepatoma cell line was donated by Dr. Chiba K (Mitsubishi Tanabe Pharma). These cells and human HEK293F (Invitrogen) were cultured in RD medium^17^ containing an equal volume of DMEM and RPMI1640 with heat‐inactivated 7% FBS (Invitrogen) in a humidified incubator (5% CO_2_) at 37°C. Aseptic processing was strictly controlled by air purifiers (MediAir, Pieras Co., Ltd.).

### 2.3 Primary antibodies, growth factors, and inhibitors

All rat mAb used in the present study were produced by Masuko T, et al.^18^, ^19^, ^20^, ^21^, ^22^, ^23^, ^24^, ^25^, ^26^ F344 rats were immunized three to six times with RH7777 rat hepatoma cells expressing human target proteins fused to GFP. Splenocytes from immunized rats were fused with P3X63Ag8.653 cells, and cloned hybridoma cells secreting mAb against target proteins were established. These mAb reacted with RH7777 transfectants expressing GFP‐fused target proteins in a GFP intensity‐dependent manner.^27^, ^28^, ^29^ Rat mAb recognizing HER1 (Ab83‐3), HER2 (Ab6‐5), HER3 (Ab1 and Ab4), HER4 (P6‐1), MET (Ab57), L‐type amino acid transporter 1/LAT1 (Ab1‐27), cationic amino acid transporter 1/CAT1 (CA2), alanine‐serine‐cysteine transporter/ASCT2 (Ab3‐8 and Ab3‐7), system x^−^_c_ transporter/xCT (Ab 2–31 and Ab2‐25), epithelial cell adhesion molecule/EpCAM (1D12), standard CD44/CD44s (RV7), variant CD44/CD44v8 (RV14), CD44v9 (RV3), and CD98 heavy chain (CD98hc, HR35) of human cells were used. Regarding HER3,^30^, ^31^ ASCT2,^25^ and xCT,^21^, ^22^, ^23^ two mAb recognizing different epitopes were used. patritumab (U3‐1287),^18^, ^32^ a fully human mAb, was kindly donated by Daiichi Sankyo Company (Tokyo, Japan). Pertuzumab (Perjeta®), a humanized mAb, was obtained from Roche‐Chugai (Tokyo, Japan). Rabbit mAb or polyclonal antibodies (pAb) against p44/42 MAPK (ERK1/2, #9102), phospho (Thr202/Tyr204)‐p44/42 MAPK (ERK1/2, #9101), AKT (#9272), phospho (Ser473)‐AKT (#8200), HER1 (#4267), HER2 (#2165), HER3 (#12708), phospho‐HER3 (#4791), MET (#8198), phospho‐MET (#3077), FOXM1 (#5436), and GAPDH (#2118) were obtained from Cell Signaling Technology (CST). Anti‐HER3 mouse mAb (#sc‐7390, Santa Cruz, CA, USA) was also used. NRG1 (CYT‐407, ProSpec), and HGF (PHG0254, Invitrogen) were added to the cell culture with FBS‐free RD medium. The tyrosine kinase inhibitor for MET, PHA665752 (JPY21500) was obtained from Selleck Bioteck. U0126 was obtained from Wako Pure Chemical Industries. Thiostrepton was obtained from R&D Systems.

### 2.4 FCM

Cells (2.0 × 10^5^) were mixed with the primary rat mAb (10 μg/mL) on ice for 1 h and then incubated with 50 μL of the phycoerithrin (PE)‐labeled donkey anti‐rat IgG (H+L) secondary antibody (1:400, #712‐116‐153, Jackson ImmunoResearch, West Grove, PA, USA) at 4°C for 1 h. Cells were washed and suspended in 0.2% BSA‐PBS and filtered through a nylon mesh (40 μm, BD Falcon). BD LSR Fortessa (Becton Dickinson) were used to perform FCM. The subtracted mean fluorescence intensity (ΔMFI) or signal‐to‐noise ratio (S/N) was calculated to quantify cell‐surface protein expression.

### 2.5 Immunohistochemistry (IHC) of human colon tissue sections

Colon adenocarcinoma with a matched adjacent colon tissue array (CO487, US Biomax, Inc.) was used. Following deparaffinization and rehydration, tissue sections were treated with solution (Target Retrieval solution S1699) at 98°C for 5 min 3 times by a microwave to retrieve the antigen. After three washes with PBS (“washing” hereafter), these sections were incubated with 3% H_2_O_2_–methanol at room temperature for 10 min to quench the effects of endogenous peroxidase activity. After washing, they were incubated with Block Ace (DS Pharma Biomedical Co., Ltd.) at room temperature for 2 h. They were then incubated with Ab4 anti‐HER3 (10 μg/mL) or Ab57 anti‐MET mAb (10 μg/mL) primary rat mAb diluted in 1% BSA‐PBS at 4°C overnight. After washing, sections were incubated with biotinylated goat anti‐rat IgG (Vector Laboratories) diluted 1:200 in 2% BSA‐PBS at room temperature for 30 min. After washing, samples were treated with avidin‐biotin‐peroxidase complex (ABC) reagent (Vector Laboratories) diluted 1:50 in 2% BSA‐PBS at room temperature for 30 min. After washing, sections were incubated with 0.05% 3,3′‐diaminobenzidine (Dojin Chemicals) and 0.01% H_2_O_2_ in 0.1 M Tris–HCl (pH 7.4), and then counterstained with hematoxylin. Sections were dehydrated, cleared in xylene, and mounted in Permount (Fisher Scientific). IHC scores for HER3 or MET expression were set based on the standard HER2 test. IHC gave a score of 0–3, indicating the amount of HER3/MET in tissue samples. HER3/MET expression in normal (adjacent to or distant from CRC) and cancer tissues was estimated as score 0 (negative), score 1 (weak or borderline), score 2 (intermediate), or score 3 (strong) by two pathologists.

### 2.6 Immunoprecipitation (IP)

Cells were solubilized by lysis buffer (150 mM NaCl, 50 mM Tris (pH 7.4), 1% Nonidet P‐40), and protease inhibitor cocktail (#03969–21, Nacalai, Kyoto, Japan) at 4°C for 20 min. Cell lysates were reacted with anti‐MET or anti‐HER2 mAb (10 μg/mL) at 4°C overnight. Antibody‐bound proteins were mixed with Protein G Sepharose 4 Fast Flow (50% slurry, GE Healthcare) for 2 h, and beads were collected by centrifugation at 9000 × *g* for 1 min. Beads were washed 3 times with lysis buffer and once with IP wash buffer (50 mM Tris (pH 8.0)). SDS sample buffer (20% glycerol, 2% SDS, 0.02% Bromophenol blue, and 100 mM DTT) was added to the beads and heat treated at 95°C for 4 min. The supernatant, separated by centrifugation at 10000 × *g* for 1 min was used for Western blot (WB).

### 2.7 WB

Whole cell extracts were prepared using lysate buffer (50 mM Tris–HCl (pH 7.4), 150 mM NaCl, 1% NP40, and 0.1% SDS) with a Protease and Phosphatase Inhibitor Cocktail (Nacalai). Protein concentrations were measured using the BCA assay (Takara Bio, Shiga, Japan). Samples were diluted in 5 × SDS buffer (0.225 M Tris–HCl, 50% glycerol, 5% SDS, 0.05% BPB, and 0.25 M DTT) and treated at 95°C for 5 min. Proteins (20 μg/lane) were separated by 8 ~ 10% SDS‐PAGE and transferred onto polyvinylidene fluoride (PVDF) membranes. Non‐specific binding sites on PVDF membranes were blocked with 5% Skim milk (Yukijirushi) for 1 h. Membranes were incubated with antibodies against HER3, MET, phospho‐HER3, phospho‐MET, phospho‐ERK, ERK, FOXM1, and GAPDH (CST) for 16 h, and then with horseradish peroxidase‐labeled goat anti‐rabbit or mouse IgG (H+L) pAb (1:400, #115‐035‐144 or #115‐035‐062, Jackson ImmunoResearch). Immunoreactive proteins were visualized by an ECL system (ImageQuant RT ECL, GE Healthcare) using Chemi‐Lumi One Super (Nacalai) in Kindai University, and by exposing X‐ray film (Super RX, Fujifilm, Tokyo, Japan) using a detection solution (Hi‐RENDOL, Hi‐RENFIX, Fujifilm) or by an ECL system (ChemiDoc, BioRad, Hercules) in Tohoku University.

### 2.8 Establishment of KO cell lines

KO was performed as recently described^18^, ^25^, ^33^ using pX330 and pCAG‐EGxxFP^34^ purchased from Addgene. In CRISPR/Cas9‐based gene disruption, guide (g) RNA sequences (5’‐AGCTGTGGCAGCGTCAACAG‐3′) corresponding to the MET gene (394–413 bp from the initiation ATG site), (5’‐GAGGGCGAACGACGCTCTGC‐3′) HER3 gene (3–22 bp from the initiation ATG site), and FOXM1(5’‐CCGTCGGCCACTGATTCTCA‐3′) gene (15–33 bp from the initiation ATG site) were designed using CRISPR direct (https://crispr.dbcls.jp/). SW1116 cells were used to generate HER3 and/or MET and the FOXM1‐KO cell line using the pX330 (Addgene) and pCAG‐EG × ×FP (Addgene) CRISPR/Cas9 vectors. The gene‐specific region of gRNA sequences was designed by the CRISPR design tool from CRISPR direct (https:/crispr.dbcls.jp/). Single clones were picked up and the KO efficiency was assessed by WB and FCM. Cells were seeded onto 35‐mm dishes (BD BioCoat, Franklin Lakes, NJ, USA) in 1 mL of RD medium, and plasmid DNA (5 μg) was introduced into cells of approximately 80% confluency using Xfect transfection reagent (Takara Bio Inc.). The co‐transfection of pX330 and pUC19 (#3219, Takara Bio) containing the puromycin‐resistant gene was also performed, and cells were cultured with puromycin (Invitrogen, 2 μg/mL) for 10 days.

### 2.9 Cell cycle analysis

The cell cycle distribution was analyzed by FCM. Briefly, cells were incubated for 96 h, washed with cold PBS, fixed in 70% ethanol overnight at −20°C, and stained with DAPI (Nacalai) reagent at 4°C for 15 min. The fluorescence intensity of the final mixture was analyzed by BD LSR Fortessa.

### 2.10 Cell proliferation analysis

Cell proliferation was assessed by WST‐8 (Cell Count Reagent SF, Nacalai). After cells were cultured in 96‐well plates, WST‐8 (10 μL /well) was added to each well at different time points and incubated at 37°C for 2 h. Absorbance (measurement: 450 nm/reference: 600 nm) was measured using a fluorescence spectrophotometer (Infinite 200 PRO series; Tecan, Switzerland).

### 2.11 In vivo tumor therapy using the xenograft model

SW1116 (5 × 10^6^ cells) in 0.2 mL of PBS were s.c. injected into the flank of KSN mice, and visible tumors were confirmed in all mice. At this point (Day 1), patritumab, PHA665752, the combination of patritumab and PHA665752, or PBS as a control was i.p. injected, followed by three additional injections on Days 7, 13, and 19. WT, HER3‐KO, MET‐KO, and HER3/MET‐double KO (dKO) SW1116 (5 × 10^6^ cells/mouse) in 0.2 mL of PBS were s.c. injected into the flank and visible tumors were confirmed in all mice. Tumor volumes were measured with a digital caliper every 3 days and quantified using the following formula: volume [mm^3^] = (length [mm]) × (width [mm])^2^ × 0.5.

### 2.12 Anchorage‐dependent growth in 3D cultures

Matrigel® (CORNING®) was added at a volume of 150 μL to 8‐well chamber slides (WATSON Bio Lab) and incubated at 37°C for 15 min, after which 5000 cells were seeded. Patritumab and the MET inhibitor were added the next day, and after a 4‐day incubation, 50 cells were measured as one colony after imaging with BIOZERO (KEYENCE) in nine fields of view/one well.

### 2.13 Statistical analysis, heat map analysis, principal component analysis (PCA), and assessment of additive or synergistic effects

Regarding the expression levels of cell surface proteins, S/N or ΔMFI was calculated. The results obtained were transformed into log_2_ values and subtracted from the median. A clustered heat map analysis and PCA were conducted with the R Project for Statistical Computing (https://www.r‐project.org/). Data for correlations were analyzed using Pearson's correlation coefficient. Experimental data were analyzed using Prism 5 for Win (GraphPad Software). The criterion for significance was at least.

*p* < 0 0.05. Combined effects by mAb or inhibitors were considered to be additive (#) or synergistic (##), as described elsewhere.^35^

## 3 RESULTS

### 3.1 Comprehensive cell‐surface protein analysis of human colon cell lines

**FIGURE 1:**
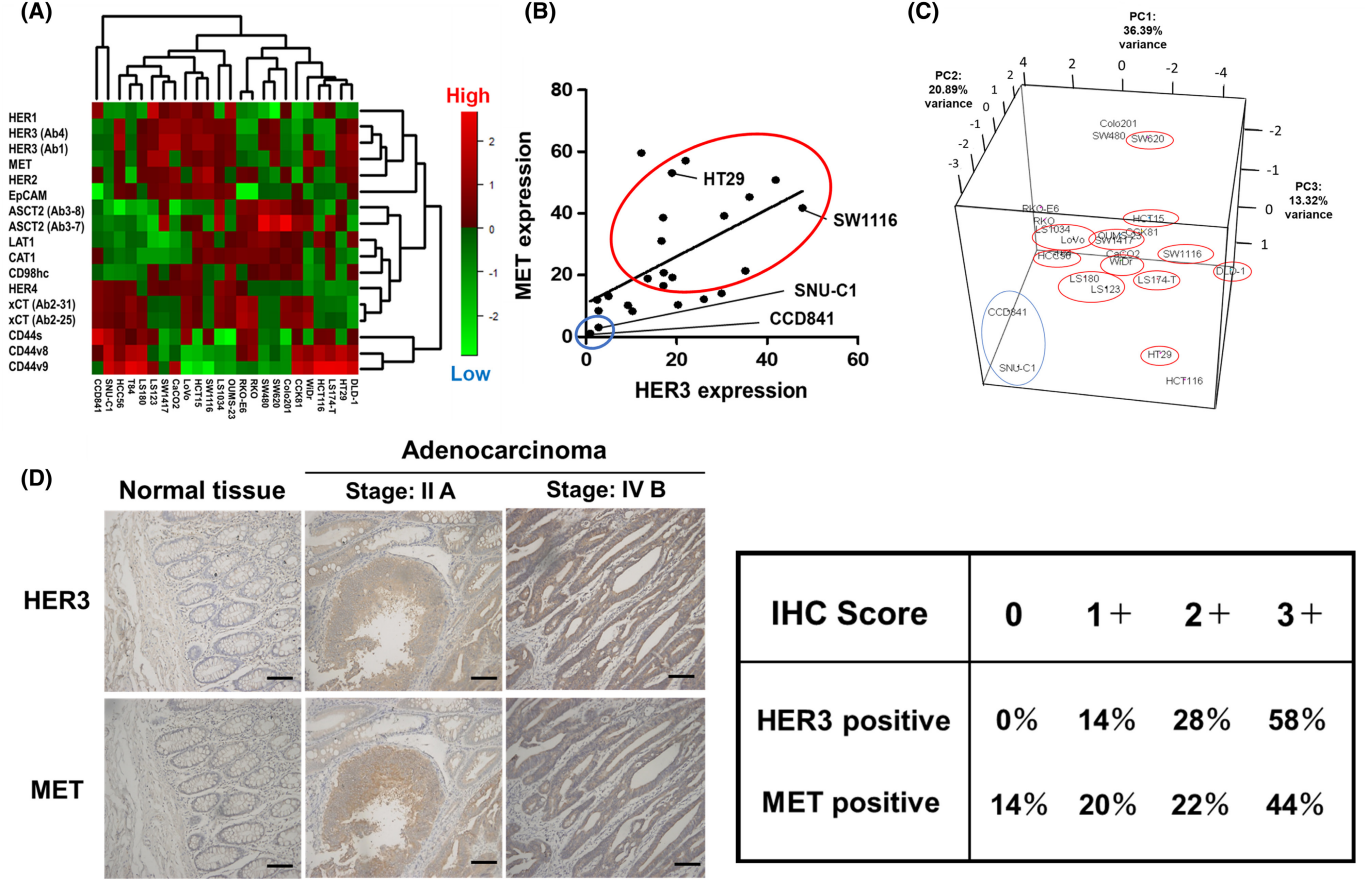
Comprehensive cell‐surface protein analysis of human CRC. (A) Heatmap of two‐way hierarchical clustering of 24 colon cell lines (columns) and 14 cell surface proteins (rows). Expression level, bright red: highest, bright green: lowest. HER3, ASCT2, and xCT were evaluated by two distinct mAb. (B) Correlation analysis of HER3 and MET expression of colon cell lines. HER3 expression levels correlated with those of MET in colon cell lines. Pearson's correlation coefficient (*r*) and *p*‐values were calculated for this analysis. MET expression versus HER3 expression rate: *r =* 0.54, *p* = 0.0065. (C) 3D‐PCA clustered cell surface expression profiling of colon cell lines. Figures (B) and (C) are highlighted by blue; HER3/MET double‐negative and by red; HER3/MET double‐high (DH). (D) Immuno‐peroxidase staining of human normal and malignant colon tissues with anti‐HER3 (upper panels) and anti‐MET (lower panels) rat mAb. Scale bar: 100 μm.

To identify targets for simultaneous inhibition, we initially examined the expression of cancer‐associated cell‐surface proteins in 24 human colon cell lines and analyzed the expression levels of 14 cell‐surface proteins with specific mAb by FCM. Protein (vertical axis)/cell (horizontal axis) types were classified by the clustered heatmap (Figure 1A). Cell lines were coarsely classified into two groups, SNU‐C1/CCD841 and the other CRC cell lines. These two cell lines have different characteristics from the other cell lines, namely, SNU‐C1 has the morphology of floating cells with round cell aggregates, and CCD841 is the only normal colon‐derived cells among the 24 colon cell lines. Surface proteins were separated into three groups. The first group included cells expressing HER1, HER2, HER3, and MET oncogene products. The second group included cells expressing ASCT2, LAT1, CAT1, CD98hc, and xCT amino acid transporters. The third group included cells expressing CD44s, CD44v8, and CD44v9 hyaluronan receptors. Cells expressing EpCAM or HER4 were located in two gaps adjacent to three groups. A positive correlation was observed between the cell‐surface expression of HER3 and MET in CRC cell lines (Figure 1B), and we estimated 13 CRC lines in 24 cell lines (54%) as a HER3/MET double‐high (DH) group. HER3 and MET protein expression was low in SNU‐C1 and CCD841 cells, uniquely characterized in Figure 1A. Furthermore, 3D‐PCA highlighted that SNU‐C1/CCD841 and the other cell lines clustered separately (Figure 1C). The higher expression of both HER3 and MET in some human CRC tissues than in normal colon tissues was demonstrated by IHC (Figure 1D). The HER3 or MET‐high (IHC score: 2 and 3) ratio was 86 or 66%, respectively, and the HER3/MET‐DH group was estimated as more than half of the CRC lines. These results indicated that an analysis of cell‐surface protein expression has the potential to classify CRC, and the HER3/MET status may extract a unique population in human CRC. We selected SW1116 as a representative HER3/MET‐high CRC in this study, because this cell line was the most suitable CRC for the establishment of KO cell lines by CRISPR/Cas9‐based gene disruption. Therefore, we used mainly SW1116 in subsequent analyses. We also used HT29 as the second HER3/MET‐DH CRC (Figure 1A–C) in some experiments.

### 3.2 Effects of NRG1 and HGF on the proliferation of SW1116 cells

**FIGURE 2:**
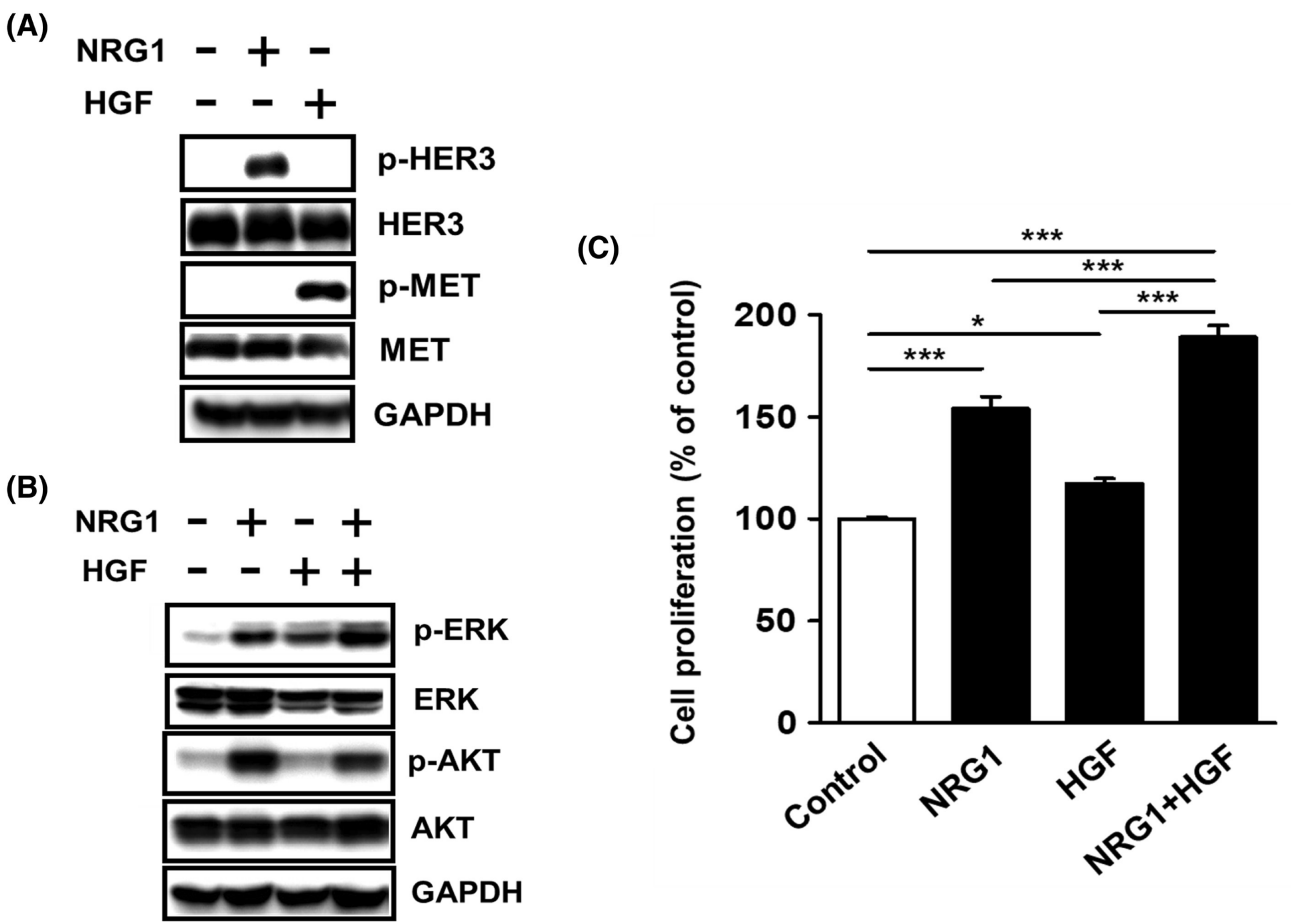
Effects of NRG1 and HGF on the phenotype of SW1116 cells. (A) SW1116 cells were treated with NRG1 (100 ng/mL) or HGF (100 ng/mL) for 5 min, and whole cell lysates (WCL) were subjected to WB. (B) SW1116 were treated with NRG1 (100 ng/mL) and/or HGF (100 ng/mL) for 36 h, and WCL were subjected to WB. (C) SW1116 (3.0 × 103 cells) were cultured in each well of a 96‐well plate for 12 h and were then treated with NRG1 (100 ng/mL) and/or HGF (100 ng/mL) in serum‐free medium for 36 h. Cell viability was assessed by the WST‐8 assay. Results were expressed as the means ± SEM (*n* = 3). *p < 0.05, ***p < 0.001, one‐way ANOVA followed by Tukey's post hoc multiple comparison test. (A) and (B) NRG1 and/or HGF were added after serum starvation for 12 h. (A) and (B) GAPDH was used as a loading control.

We examined the effects of NRG1 and HGF on the phosphorylation of HER3 and MET and on the proliferation of SW1116 cells. A brief ligand stimulation resulted in the phosphorylation of the receptor for each ligand (Figure 2A). After a prolonged ligand stimulation, the potential activation of additional downstream signaling was verified. The phosphorylation of AKT was only induced by the NRG1 stimulation and was not further enhanced by the combined use of HGF. On the contrary, the phosphorylation of ERK was enhanced by the combined dual stimulation than by the single stimulation (Figure 2B). Furthermore, the combined dual stimulation with NRG1 and HGF significantly enhanced cell proliferation more than the stimulation with each ligand alone (Figure 2C).

### 3.3 NRG1‐induced MET phosphorylation in human CRC cells

**FIGURE 3:**
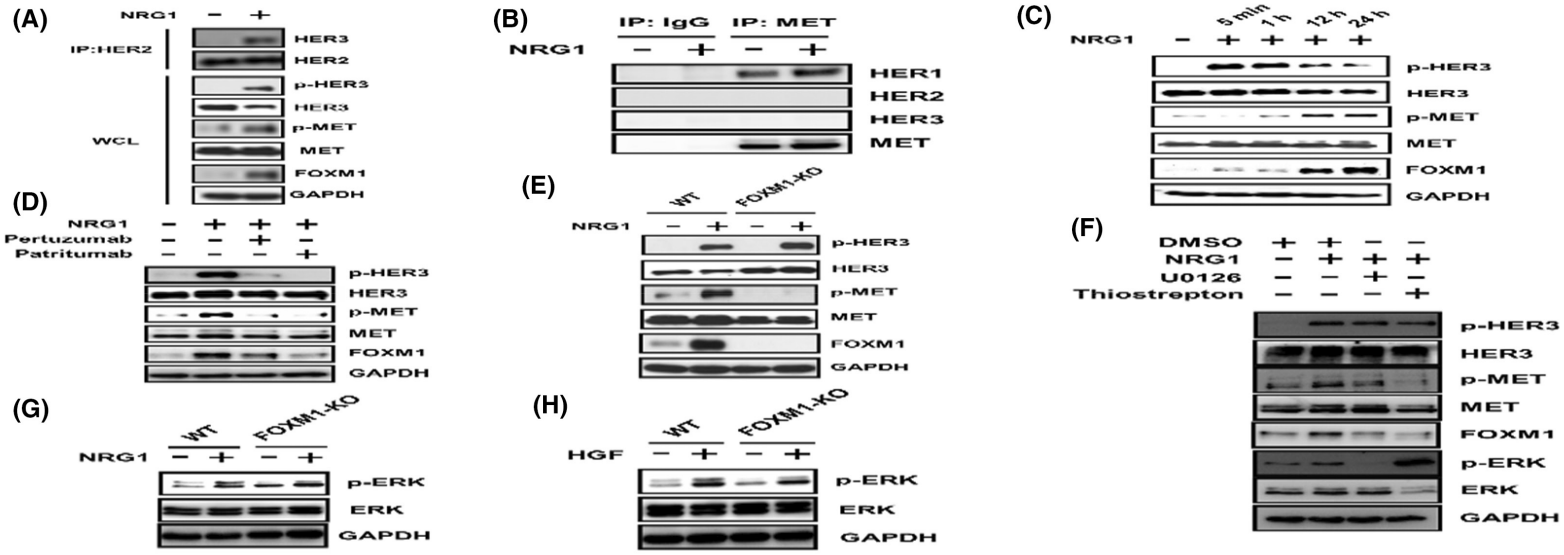
NRG1‐induced MET phosphorylation in SW1116 cells. (A) SW1116 cells were treated with NRG1 (5 ng/mL) for 12 h, and WCL or immunoprecipitates with anti‐HER2 mAb were subjected to WB. (B) SW1116 were treated with NRG1 (5 ng/mL) for 12 h, and immunoprecipitates with control IgG or anti‐MET mAb were subjected to WB. (C) Time course of MET activation by NRG1 in SW1116. SW1116 were stimulated with NRG1 (5 ng/mL). (D) SW1116 were treated with pertuzumab (10 μg/mL) or patritumab (10 μg/mL) for 1 h, followed by NRG1 (5 ng/mL) for 12 h. (E) Parent and FOXM1‐KO SW1116 were treated with NRG1 (5 ng/mL) for 12 h, and WCL were subjected to WB. (F) SW1116 were treated with U0126 (1 μM) or thiostrepton (10 μM) for 1 h, followed by NRG1 (5 ng/mL) for 12 h. (G) Parent and FOXM1‐KO SW1116 were treated with NRG1 (5 ng/mL) for 12 h. (H) Parent and FOXM1‐KO SW1116 were treated with HGF (50 ng/mL) for 12 h. NRG1 and HGF were added after serum starvation for 12 h. (A), (C–H), GAPDH was used as a loading control. (C–H), WCL were subjected to WB.

To analyze potential crosstalk between HER3 and MET, we initially examined MET activation by NRG1 in SW1116 cells. NRG1 induces the formation of the HER2/HER3 heterodimer and the activation of HER3. We confirmed the formation of HER2/HER3 heterodimers and demonstrated MET phosphorylation by the NRG1 stimulation for 12 h (Figure 3A). MET phosphorylation by the prolonged (12 h) NRG1 stimulation was also observed in HT29 cells (Figure S1). To elucidate the mechanisms underlying NRG1‐induced MET phosphorylation, we examined whether a direct interaction occurred between HER3 and MET. Heterodimers between MET and HER2 or MET and HER3 were not detected, whereas MET and HER1 formed heterodimers with or without NRG1 (Figure 3B). We then focused on FOXM1, a transcription factor that regulates MET expression.^36^ HER3 phosphorylation was induced 5 min after the addition of NRG1, whereas MET phosphorylation was only weakly observed at this point. The prominent phosphorylation of MET and induction of FOXM1 were detected 12 h after the addition of NRG1 and were maintained at least until 24 h (Figure 3C). Although total MET expression seems unchanged by FOXM1 induction in Figure 3C, however, elevation of the cell‐surface MET by NRG1 was observed with FCM analysis (Figure S2). Since the formation of HER2/HER3 heterodimer was demonstrated in the presence of NRG1 (Figure 3A), effects of anti‐HER2 pertuzumab^37^ or anti‐HER3 patritumab,^32^ which respectively recognizes HER2‐epitope or HER3‐epitope and inhibits heterodimerization between HER2 and HER3, on the NRG1‐induced HER3 activation (phosphorylation) and the induction of FOXM1, were analyzed. Pertuzumab and patritumab inhibited the NRG1‐induced phosphorylation of HER3 and MET and suppressed increases in the expression of FOXM1 (Figure 3D). FOXM1‐KO SW1116 cells were then established to investigate the contribution of FOXM1 to the NRG1‐induced phosphorylation of MET. In FOXM1‐KO SW1116, NRG1 did not induce the phosphorylation of MET, whereas the phosphorylation of HER3 was induced by NRG1 (Figure 3E). We used the MEK inhibitor (U0126) and FOXM1 inhibitor (thiostrepton) to examine the effects of MET phosphorylation induced by the NRG1 stimulation on the MAPK pathway. The NRG1‐induced phosphorylation of MET was inhibited by U0126 or thiostrepton. The inhibition of the MAPK pathway by U0126 was previously shown to decrease FOXM1 expression,^38^, ^39^ which is consistent with the present results (Figure 3F). We also examined the effects of NRG1 or HGF on the MAPK pathway in FOXM1‐KO SW1116 cells to demonstrate the importance of FOXM1‐mediated signaling induced by the NRG1 stimulation. The NRG1 or HGF stimulation induced the phosphorylation of ERK in FOXM1‐KO SW1116 cells (Figure 3G,H).

### 3.4 Effects of HER3‐ and/or MET‐KO on the cell proliferation, cell‐cycle progression, and tumor growth of CRC

**FIGURE 4:**
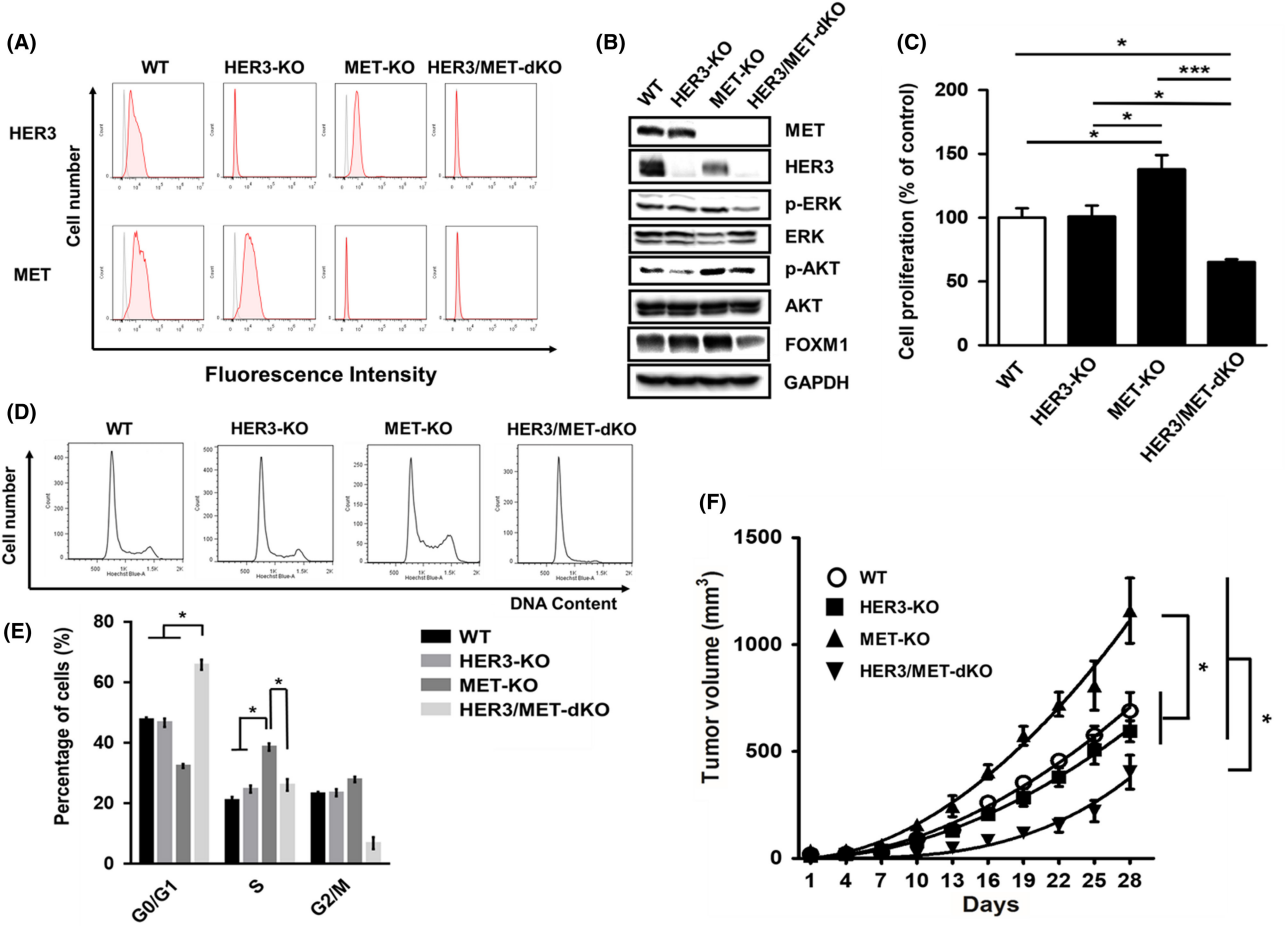
Effects of HER3‐ and/or MET‐KO on the cell proliferation, cell‐cycle progression, and tumor growth of SW1116. (A) FCM analysis of HER3 and MET expression in parent and HER3‐KO, MET‐KO, and HER3/MET‐dKO SW1116 cells. (B) WB analysis of HER3 and MET expression in parent and HER3‐KO, MET‐KO, and HER3/MET‐dKO SW1116 cells. Whole cell lysates were subjected to WB and GAPDH was used as the loading control. (C) Cells was cultured in 96‐well plates (2000 cells/well) for 5 days. Cells were cultured with no ligand in this experiment. Cell viability was assessed with the WST‐8 assay, and results were expressed as the means ± SEM (*n* = 4). *p < 0.05, ***p < 0.001, one‐way ANOVA followed by Tukey's post hoc multiple comparison tests. (D) Cell cycle analysis by FCM with DAPI. (E) Quantification of cell cycle populations. Results are expressed as means ± SEM (*n* = 3). *p < 0.05, one‐way ANOVA followed by Tukey's post hoc multiple comparison tests. (F) Nude mice were inoculated with the indicated cells. Tumor volumes were measured every 3 days using digital calipers and quantified using the formula: volume [mm^3^] = (length [mm]) × (width [mm])^2^ × 0.5. Results are expressed as means ± SEM (*n* = 5–6). *p < 0.05, two‐way ANOVA followed by Tukey's post hoc multiple comparison tests. Cells were cultured with no ligand in this experiment.

To analyze the effects of HER3‐ and/or MET‐KO on cell growth, we established HER3 and/or MET‐KO SW1116 cells. Using FCM and WB analyses, we confirmed that HER3 proteins disappeared in both HER3‐KO and HER3/MET‐double KO (dKO) cells, whereas MET proteins disappeared in both MET‐KO and HER3/MET‐dKO cells (Figure 4A,B). ERK phosphorylation was lower in HER3/MET‐dKO cells than in wild‐type (WT), HER3‐KO, and MET‐KO SW1116 cells (Figure 4B). Additionally, FOXM1 expression in HER3/MET‐dKO SW1116 cells was significantly decreased, as compared to HER3‐KO or MET‐KO, suggesting the role of both HER3 and MET in the regulation of FOXM1 expression. Cell proliferation was significantly lower in HER3/MET‐dKO cells than in other SW1116 cells (Figure 4C). We then examined the effects of HER3‐, MET‐, or HER3/MET‐KO on the cell‐cycle progression of SW1116 cells by FCM. In HER3/MET‐dKO SW1116 cells, cell‐cycle arrest was observed in the G0/G1 phase (Figure 4D,E). In MET‐KO cells that showed enhanced cell proliferation in Figure 4C, increases in the S‐phase fraction and decreases in the G0/G1‐phase fraction were observed (Figure 4D,E). We also investigated the effects of HER3‐ and/or MET‐KO on in vivo tumor growth by xenografted SW1116 cells in nude mice. Periodic calculation of growing tumor volumes revealed that tumor growth was strongly inhibited in HER3/MET‐dKO SW1116 tumors, as compared to parental SW1116 tumors (Figure 4F). From photo image of each excised tumor in four groups at day 28, we also obtained similar result as in Figure 4F (Data not shown).

### 3.5 Effects of patritumab and PHA665752 on the in vitro cell proliferation and in vivo tumor growth of human CRC

**FIGURE 5:**
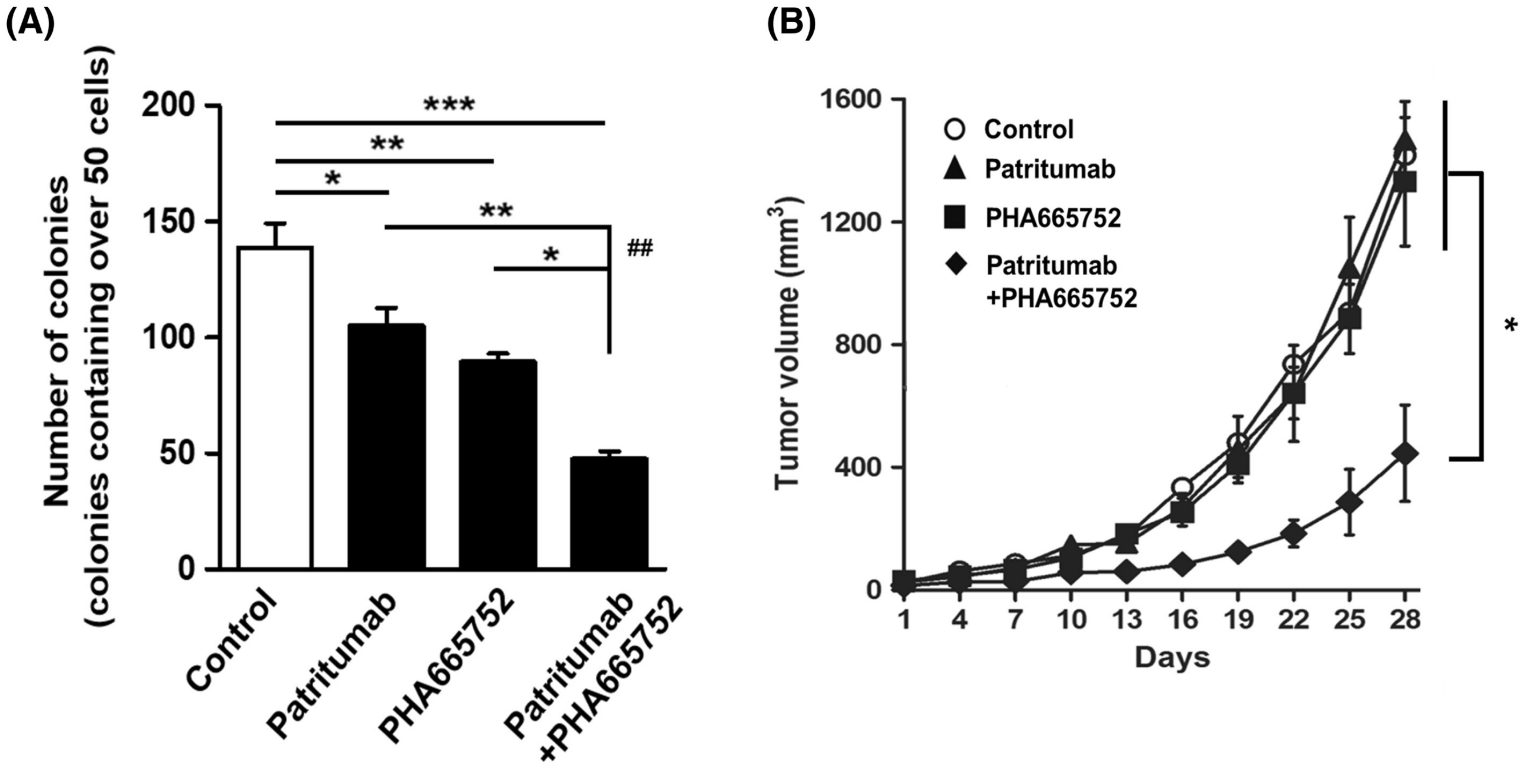
Effects of patritumab and PHA665752 on the in vitro cell proliferation and in vivo tumor growth of human CRC cells. (A) SW1116 were cultured in Matrigel‐coated slides in the presence of patritumab (30 μg/mL) and/or PHA665752 (0.5 μM) for 4 days. Cells were cultured with no ligand in this experiment. Using colonies containing more than fifty cells, results were expressed as the means ± SEM (*n* = 3). *p < 0.05, **p < 0.01, one‐way ANOVA followed by Tukey's post hoc multiple comparison tests. (B) After the confirmation of tumor development of SW1116, mice were injected i.p. with Patritumab (12 mg/kg) and/or PHA665752 (0.6 mg/kg) on days 1, 7, 13, and 19. Tumor volumes were measured every 3 days using digital calipers and were quantified using the formula: volume [mm^3^] = (length [mm]) × (width [mm])^2^ × 0.5. Results are expressed as means ± SEM (*n* = 6). *p < 0.05, two‐way ANOVA followed by Tukey's post hoc multiple comparison tests.

To examine the efficacy of the combined treatment of anti‐HER3 and anti‐MET drugs against human CRC cells, we evaluated the effects of patritumab and PHA665752 against SW1116 cells using in vitro colony formation in 3D‐culture and in vivo tumor growth in nude mice. The combined treatment of patritumab and PHA665752 resulted in the significantly stronger inhibition of colony formation (Figure 5A) and tumor growth (Figure 5B) than the single treatment with patritumab or PHA665752 alone. In view of the importance to use multiple cell lines, HT29 (HER3/MET‐high CRC) was selected for the in vitro and in vivo evaluation of dual‐targeting therapy against HER3 and MET. Although in vitro proliferation of HT29 CRC cells by singly treated PHA665752 was inhibited, inhibitory effects were significantly obvious in the combined use of patritumab and PHA665752 (Figure S3). Furthermore, patritumab and PHA665752 significantly inhibited in vivo tumor growth of HT29, as compared to singly treated patritumab or PHA665752 (Figure S4).

## 4 DISCUSSION

Due to the heterogeneity of cancer cells or cancer tissues, single‐agent therapy often resulted in limited efficacy^40^; therefore, combination therapy has become the mainstream^41^ in recent years. We have developed mAb toward effective combination therapy against specific target molecules, including oncogene products,^18^, ^19^, ^28^, ^29^, ^30^, ^31^ amino acid transporters,^20^, ^21^, ^23^, ^25^, ^26^, ^27^, ^28^, ^29^, ^33^ and adhesion molecules.^17^, ^21^, ^22^, ^24^, ^28^, ^29^ In this context, HER3 has been associated with the acquisition of drug resistance to anti‐EGFR (HER1) and anti‐HER2 cancer therapy,^11^, ^42^ and MET is involved in the proliferation, migration, invasion, and motility^43^, ^44^, ^45^ of cancer cells. Therefore, HER3 and MET have been anticipated as potential therapeutic targets. We produced novel anti‐HER3 mAb that exerted anti‐tumor effects on human colon and breast cancer cells in xenografted mouse models,^18^ and recently developed specific anti‐MET mAb^28^, ^29^ in addition to transporters^20^, ^25^, ^26^, ^28^, ^29^ and adhesion molecules.^17^, ^21^, ^28^, ^29^

In the present study, we analyzed the expression of 14 cancer‐associated proteins in 24 CRC cell lines by FCM. A positive correlation was reported between the expression of HER2 and HER3 in primary colorectal cancer.^46^ Similar results were obtained in the present study, namely, the correlation coefficient (*r*) of HER2 versus HER3 = 0.711 (data not shown). A positive correlation between the expression of HER3 and MET (Figure 1B) was noted in 24 human colon cancer cell lines (*r* = 0.54), but not in 25 breast and 22 lung cancer cell lines (*r* = 0.01 in breast cancers, and *r* = 0.27 in lung cancers) (data not shown).

Regarding the effects of HER3 and MET on cancer cell proliferation, the combined stimulation of both ligands enhanced cell proliferation more than either ligand alone (Figure 2C), suggesting that HER3 and MET contribute to cell proliferation in a concerted manner. Since NRG1 induces not only HER3 phosphorylation but also MET phosphorylation (Figure 3A), a pathway in which HER3 regulates MET signaling in CRC was postulated. The formation of the HER3/MET heterodimer was reported in human lung cancer cells.^47^ We did not detect a direct relationship between HER3 and MET; however, HER1/MET heterodimers were identified in the presence or absence of NRG1 (Figure 3B). The phosphorylation of MET, but not HER3, and the induction of FOXM1 were diminished in FOXM1‐KO SW1116 cells (Figure 3E), indicating that FOXM1 is essential for the NRG1‐induced phosphorylation of MET. In this context, Figure 3G and 3H shows that HER3 and MET can transmit FOXM1‐independent individual proliferation signals, in addition to FOXM1‐dependent HER3/MET‐mediated proliferation signal. These results suggest the existence of a pathway through which HER3 regulates MET signaling via FOXM1. Although we investigated the involvement of HGF as a mechanism for MET phosphorylation by NRG1, there was no increase in HGF expression at the mRNA or protein level in SW1116 cells from the NRG1 stimulation (data not shown).

**FIGURE 6:**
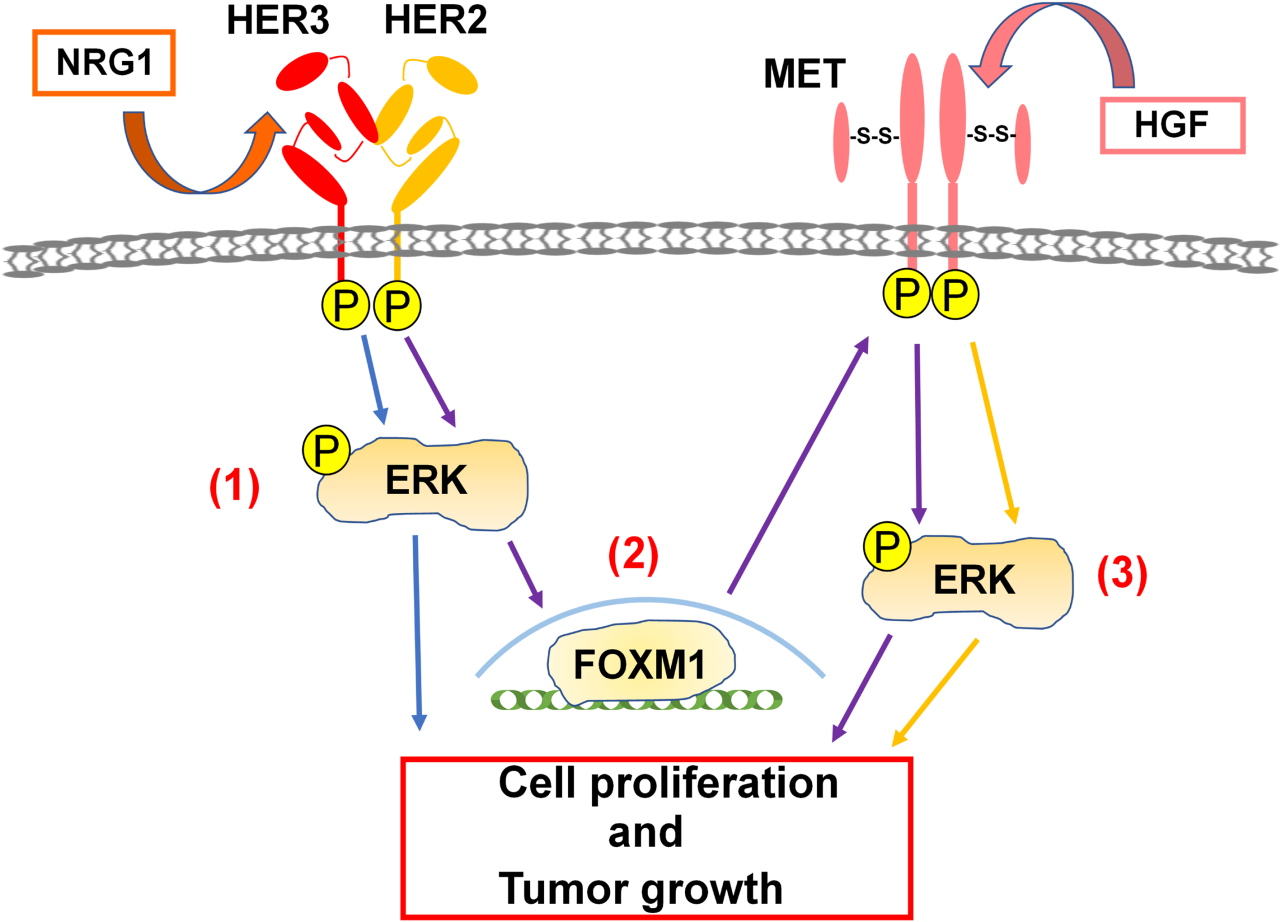
Schematic of ERK activation in CRC cells by NRG1 and HGF. We proposed three signaling pathways in CRC. (1) The FOXM1‐independent activation of the MAPK signal by a brief NRG1. stimulation of HER3 (blue arrows). (2) Activation of the MET signal via FOXM1 by a prolonged NRG1 stimulation of HER3 (purple arrows). (3) Activation of the MAPK signal by a HGF stimulation (yellow arrows).

Effects of NRG1 and HGF on colon cancer cell proliferation shown in Figures 2 and 3 are summarized in Figure 6. We assumed that at least three MAPK‐based signals may be induced by the stimulation with NRG1 and HGF. The first is the activation of FOXM1‐independent MAPK signaling by the NRG1 stimulation (Figure 3G); the second is a signal that induces the activation of MET via FOXM1 upon the NRG1 stimulation (Figure 3E), which was newly discovered in the present study; and the third is a signal that induces the activation of MAPK by the HGF stimulation (Figure 3H). The activation of these three MAPK signals enhanced the phosphorylation of ERK by the combined use of ligands, which we assume is the reason why HER3 and MET contributed to cell proliferation in a concerted manner (Figure 6).

Contrary to expectations, MET‐KO promoted in vitro cell proliferation and in vivo tumor growth of SW1116. Although a marked increase in the percentage of cells in the S phase may be involved in these phenomena, the underlying mechanisms remain unclear. In HER3/MET‐dKO SW1116 cells, in vitro cell proliferation and in vivo tumor growth were significantly inhibited and G0/G1 arrest was induced, indicating that simultaneous HER3/MET targeting was effective (Figure 4B–F). Since FOXM1‐KO markedly inhibited cell proliferation (Figure S5), decreased expression of FOXM1(Figure 4B) may be an important factor in suppressed cell proliferation (Figure 4C) and tumor growth (Figure 4F) of HER3/MET‐dKO cells.

We showed that HER3 and MET indirectly affected each other in the presence of each ligand; however, essential or ultimate effectors transcriptionally regulated by FOXM1 that are responsible for the NRG1‐induced phosphorylation of MET and anti‐tumor effects by the dual targeting of HER3/MET remains to be solved. As to possible mechanism of “NRG1‐stimulated and FOXM1‐mediated MET phosphorylation,” we remark cell‐surface expression of MET. Total MET expression seems not altered in FOXM1 induction in Figure 3C, however, elevation of the cell‐surface MET by NRG1 was observed with FCM analysis (Figure S2). It has been reported that both the proportion of MET dimers and the level of MET autophosphorylation increased at high MET protein density,^48^ therefore, possible MET homodimerization promoted by high density of cell‐surface MET in this study could result in the efficient phosphorylation of MET in SW1116 cells.

In recent years, the combination therapy has been attracting attention, because of the possible additive or synergistic potential for effective therapeutic effects through independent mechanisms of action.^49^ However, it is not easy to search for effective molecules for co‐targeting from huge combinations, as in the example of co‐targeting of IGF1R with aromatase inhibitor, estrogen receptor antagonist,^50^, ^51^ or HER2 inhibitor,^52^ which has resulted in the poor clinical response. In this context, dual targeting of HER3 or MET seems to be one of the most promising mechanisms, which could overcome the resistance of cancer cells against anti‐HER1 and anti‐HER2 therapy.^11^, ^12^, ^53^, ^54^, ^55^

The present results demonstrated that HER3 and MET were not directly associated with each other in CRC but cooperated for the cellular and tumor growth of CRC (Figure 6). The combination of anti‐HER3 (Patritumab) and anti‐MET (PHA665752) drugs was effective against HER3/MET‐high SW1116 cells, indicating the potential of the dual inhibition of HER3 and MET as the possible targeting therapy overcoming the resistance of cancer cells against anti‐HER1 and anti‐HER2 therapy in CRC and various human epithelial cancers.

## AUTHOR CONTRIBUTIONS

**Akitaka Yamasaki:** Investigation (lead); methodology (lead); writing – original draft (equal). **Rikuto Miyake:** Investigation (supporting); methodology (supporting). **Yuta Hara:** Formal analysis (supporting); investigation (supporting); methodology (supporting). **Hideki Okuno:** Investigation (supporting); methodology (supporting). **Takuya Imaida:** Investigation (supporting); methodology (supporting). **Kouki Okita:** Investigation (supporting); methodology (supporting). **Shogo Okazaki:** Formal analysis (supporting); investigation (supporting). **Yasutoshi Akiyama:** Investigation (supporting); validation (supporting). **Kenji Hirotani:** Methodology (supporting). **Yuichi Endo:** Investigation (supporting); validation (supporting). **Kazue Masuko:** Investigation (supporting); methodology (supporting); validation (supporting). **Takashi Masuko:** Conceptualization (lead); funding acquisition (lead); investigation (supporting); methodology (supporting); writing – original draft (equal); writing – review and editing (lead). **Yoshihisa Tomioka:** Conceptualization (equal); investigation (supporting); supervision (supporting).

## FUNDING INFORMATION

This work was supported by MEXT/JSPS KAKENHI Grant Number 18H05463 (Grant‐in‐Aid for Scientific Research on Innovative Areas, Toward new frontiers: encounter and synergy of state‐of‐the‐art astronomical detectors and exotic quantum beams).

## CONFLICT OF INTEREST STATEMENT

Masuko T and Endo Y were supported by commissioned research fund to Kindai University (J20084, 2020–2021) from HEALIOS K.K. (Japan). The other authors declare that they have no conflicts of interest.

## ETHICS APPROVAL

This study was approved by the Institutional Review Board of the Pharmaceutical department (approval no. 22–206), Kindai University.

## Supporting information


Figure S1:

Figure S2:

Figure S3:

Figure S4:

Figure S5:

## Data Availability

The data used and analyzed during this study are available from the corresponding author on request.
