# Supplementary figures and images for: Dual‐targeting therapy against HER3/MET in human colorectal cancers
Source: Cancer Med. 2023 Feb 7;12(8):9684–96. doi: 10.1002/cam4.5673 (PMC10166911; doi:10.1002/cam4.5673)

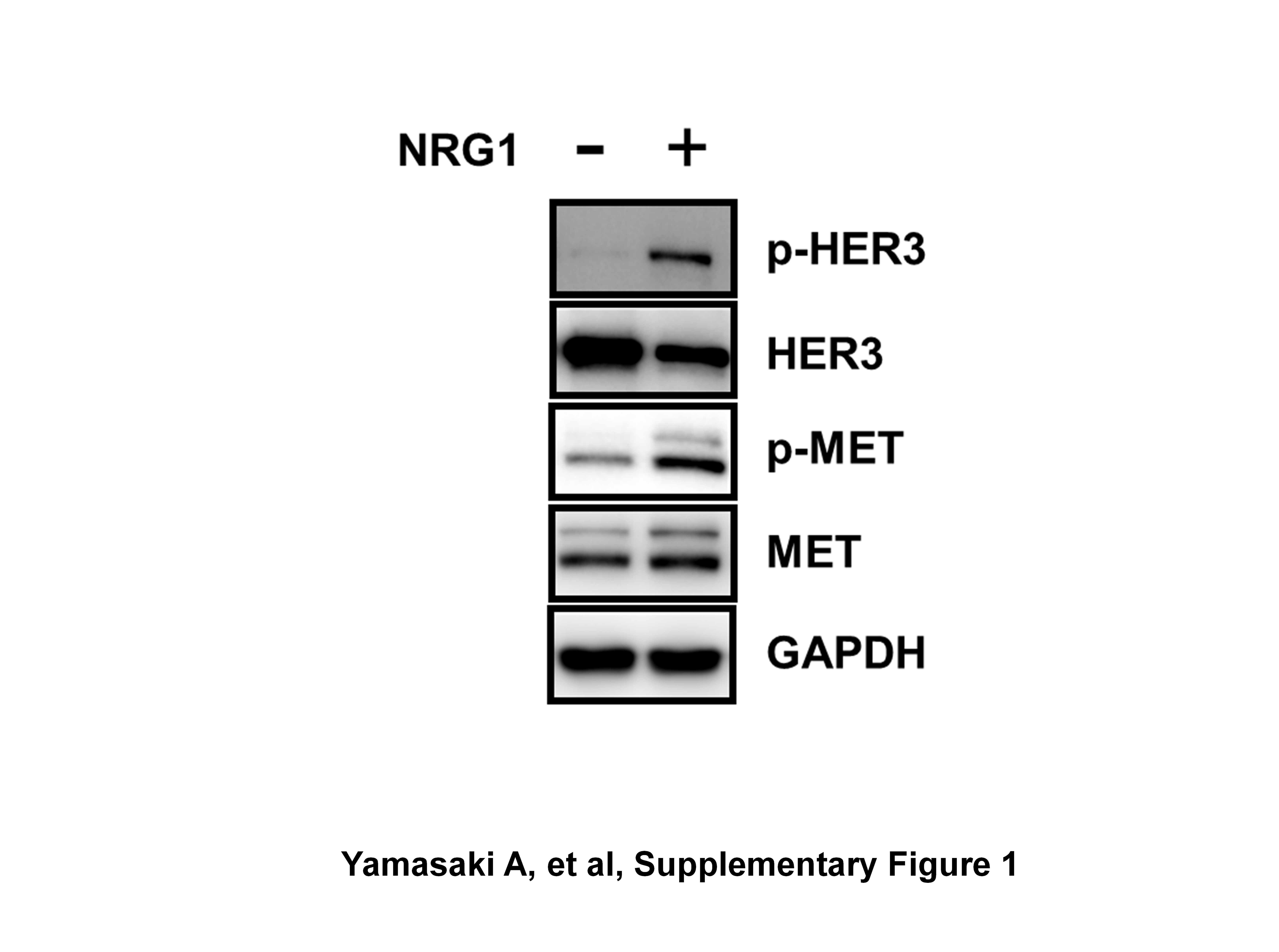

Supplement: Supplementary file 1 — Figure S1: [file CAM4-12-9684-s002.tif]

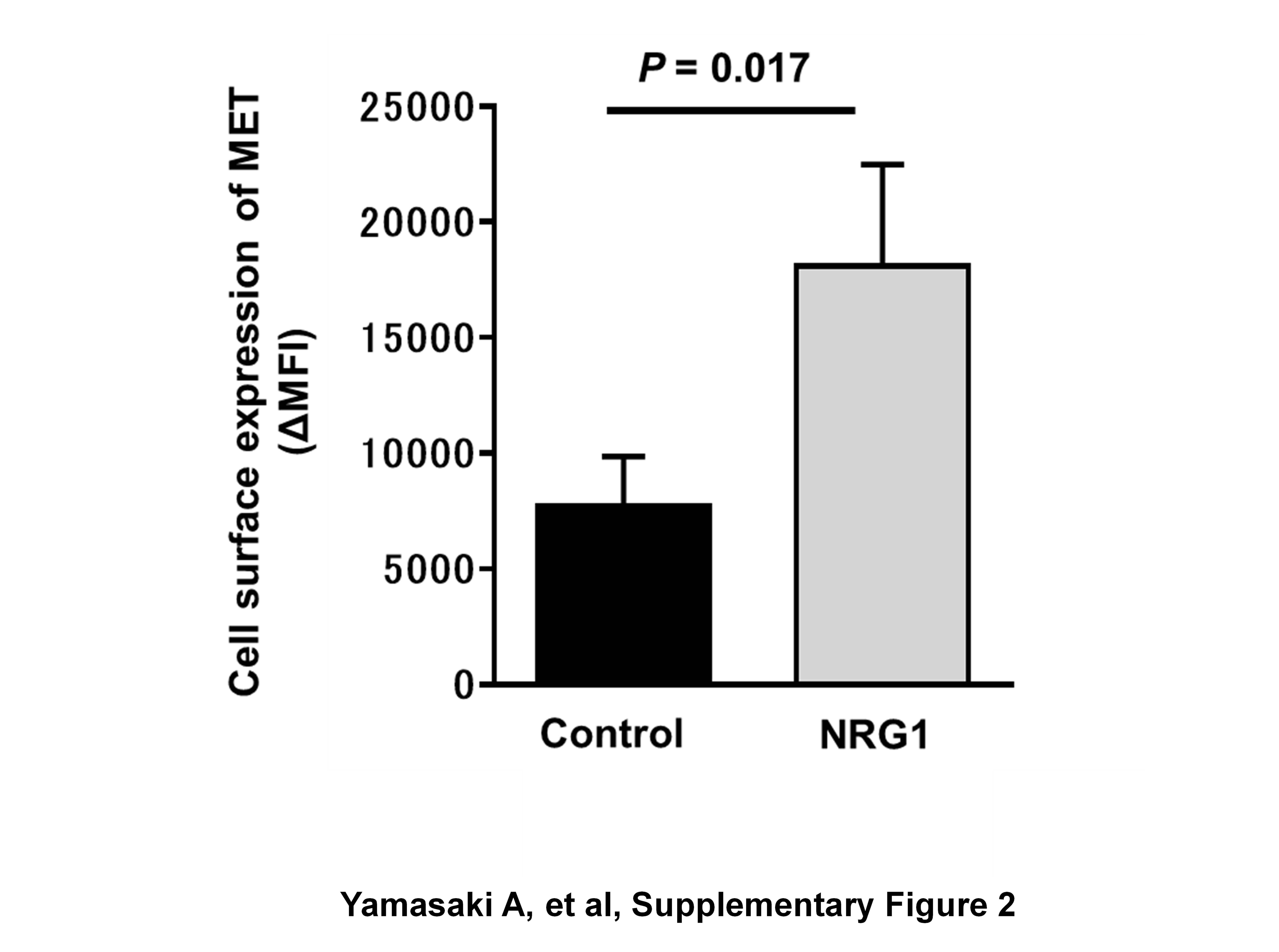

Supplement: Supplementary file 2 — Figure S2: [file CAM4-12-9684-s005.tif]

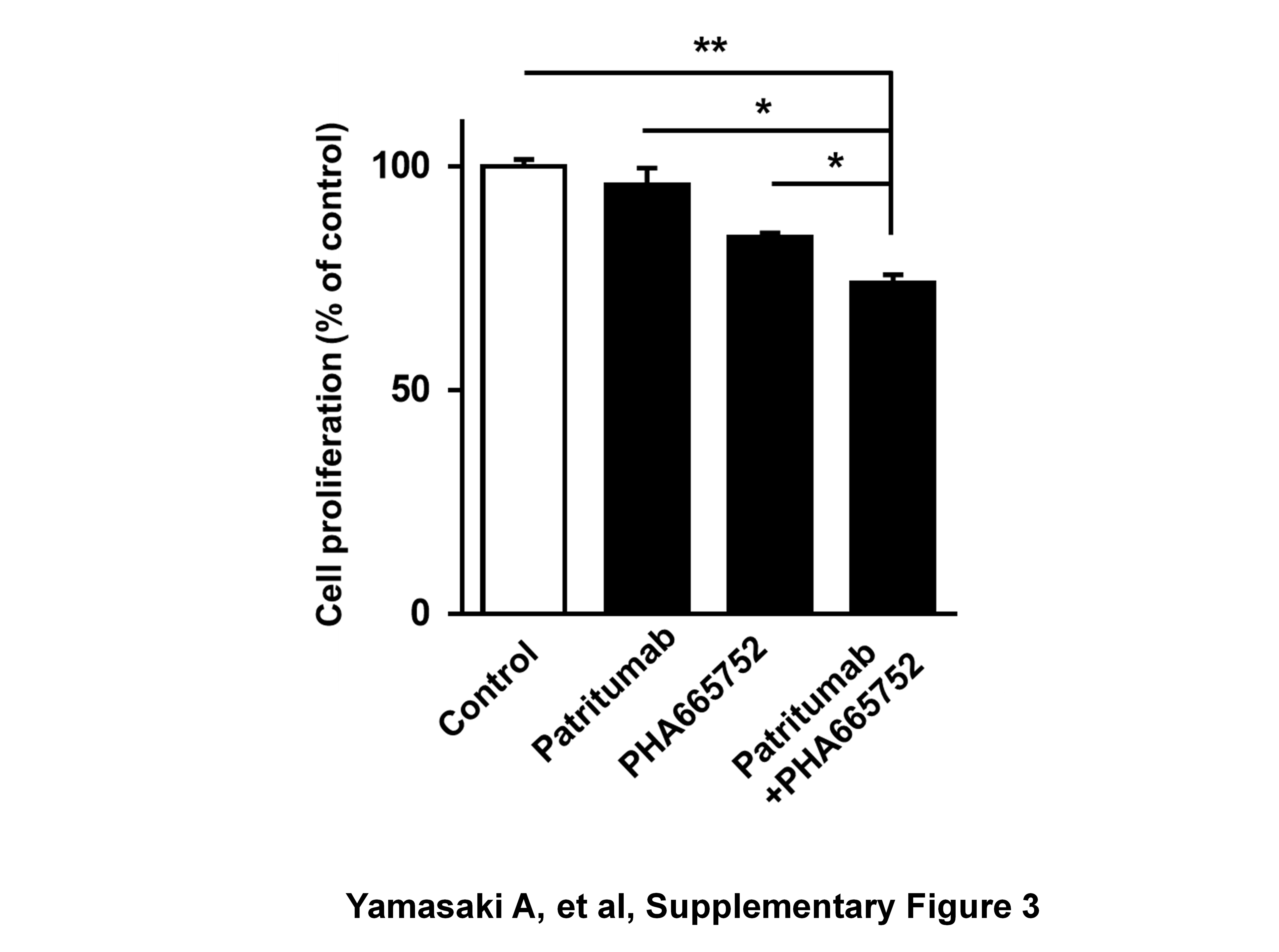

Supplement: Supplementary file 3 — Figure S3: [file CAM4-12-9684-s001.tif]

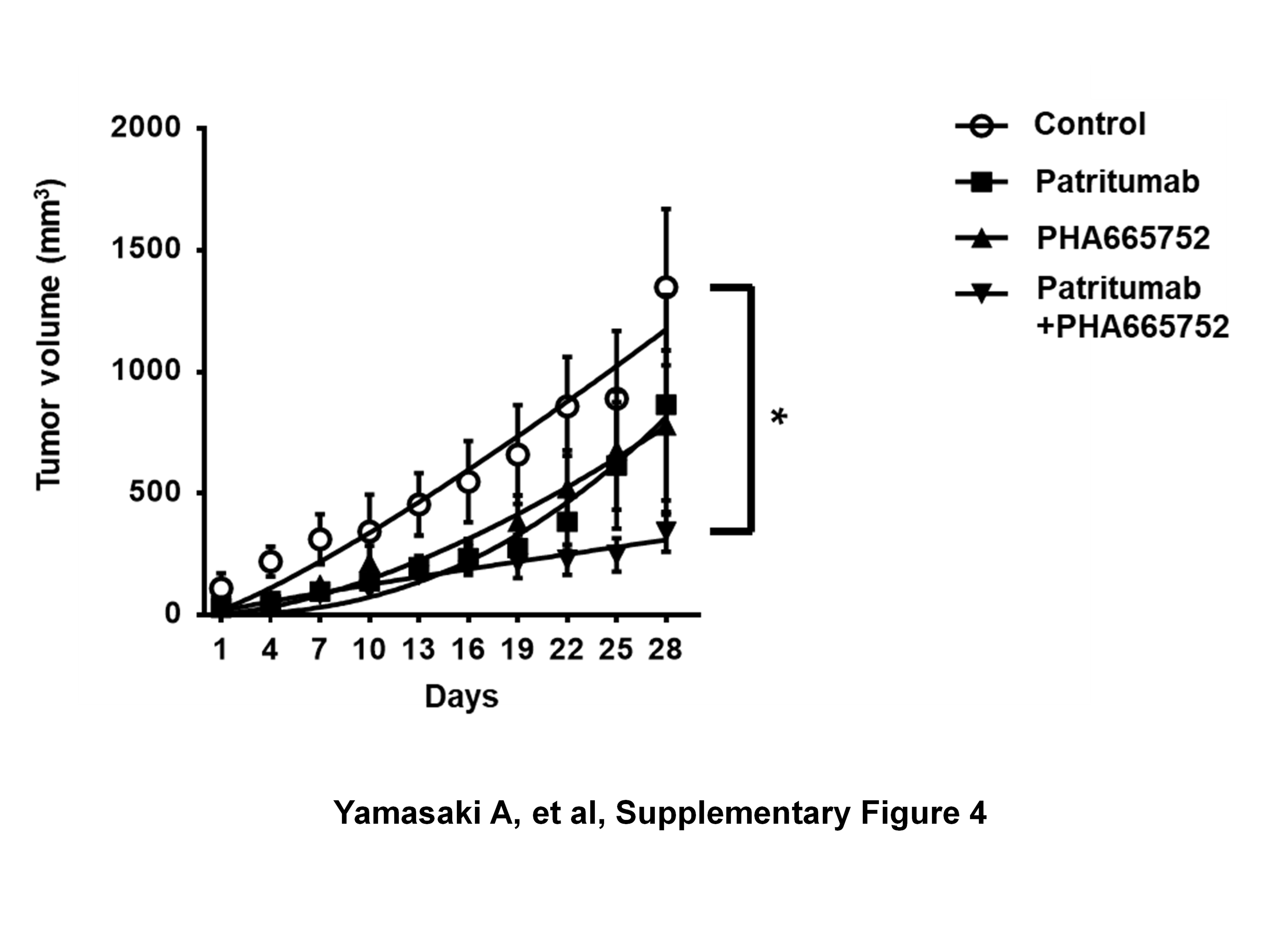

Supplement: Supplementary file 4 — Figure S4: [file CAM4-12-9684-s004.tif]

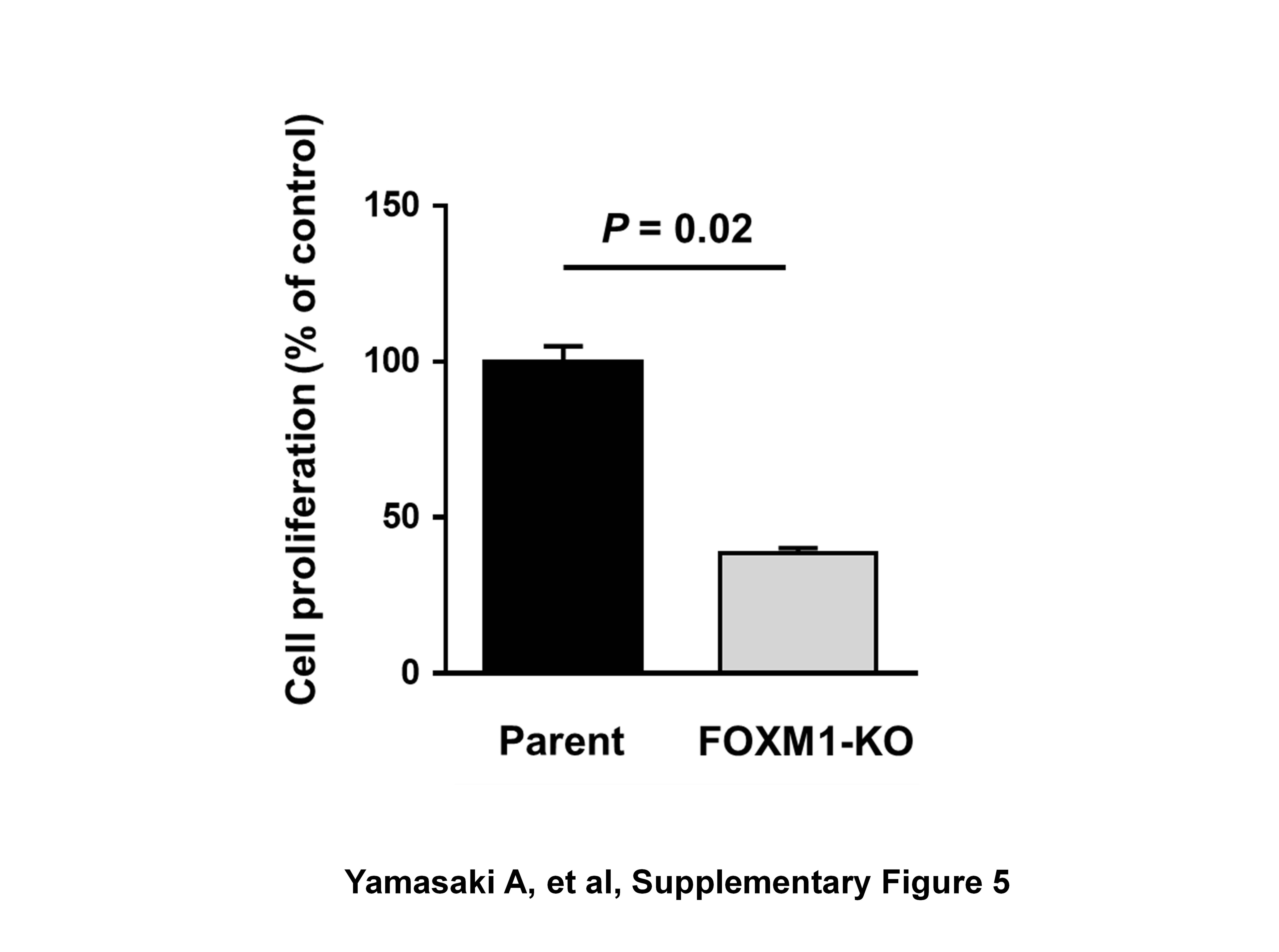

Supplement: Supplementary file 5 — Figure S5: [file CAM4-12-9684-s003.tif]
